# A VEGFR targeting peptide-drug conjugate (PDC) suppresses tumor angiogenesis in a TACE model for hepatocellular carcinoma therapy

**DOI:** 10.1038/s41420-022-01198-9

**Published:** 2022-10-06

**Authors:** Dongyuan Wang, Jiacheng Liu, Tongqiang Li, Yingliang Wang, Xiaoming Liu, Yaowei Bai, Chaoyang Wang, Shuguang Ju, Songjiang Huang, Chongtu Yang, Chen Zhou, Yu Zhang, Bin Xiong

**Affiliations:** 1 Department of Pharmacy, Union Hospital, Tongji Medical College, Huazhong University of Science and Technology, Wuhan, 430022 China; 2 Hubei Province Clinical Research Center for Precision Medicine for Critical Illness, Wuhan, 430022 China; 3 Department of Radiology, Union Hospital, Tongji Medical College, Huazhong University of Science and Technology, Wuhan, 430022 China; 4 Hubei Province Key Laboratory of Molecular Imaging, Wuhan, 430022 China; 5 Department of Interventional Radiology, The First Affiliated Hospital of Guangzhou Medical University, Guangzhou, 510120 China

**Keywords:** Targeted therapies, Drug delivery

## Abstract

Transcatheter arterial chemoembolization (TACE) has become the preferred therapy for unresectable advanced hepatocellular carcinoma (HCC). However, the embolization of tumor-feeding arteries by TACE always leads to hypoxia-related tumor angiogenesis, which limited the therapeutic effect for HCC. In this paper, we used a VEGFR targeting peptide VEGF125 − 136 (QKRKRKKSRYKS) to conjugate with a lytic peptide (KLUKLUKKLUKLUK) to form a peptide-drug conjugate (PDC). We used cell affinity assay to detect the peptide binding ability to VEGFR highly expressed cell lines, and CCK8, cell apoptosis to confirm the cellular toxicity for different cell lines. Meanwhile, we created a VX2 tumor-bearing rabbit model to assess the in vivo anti-tumor effect of the peptide conjugate in combination with TAE. HE staining was used to verify the in vivo safety of the peptide conjugate. IHC was used to assess the anti-angiogenesis and cell toxicity of the peptide conjugate in tumor tissues. The peptide conjugate could not only target VEGFR in cell surface and inhibit VEGFR function, but also have potent anti-cancer effect. We luckily found the peptide conjugate showed potent cytotoxicity for liver cancer cell Huh7 (IC50 7.3 ± 0.74 μM) and endothelial cell HUVEC (IC50 10.7 ± 0.292 μM) and induced cell apoptosis of these two cell lines. We also found the peptide conjugate inhibited cell migration of HUVEC through wound healing assay. Besides, these peptides also showed better in vivo anti-tumor effect than traditional drug DOX through TACE in VX2 rabbit tumor model, and efficiently inhibit angiogenesis in tumor tissues with good safety. In conclusion, our work may provide an alternative option for clinical HCC therapy via TACE combination.

## Abstract (web summary)

Schematic presentation of the design of VEGFR targeting peptide conjugate (QR-KLU) and the antineoplastic efficacy of peptide QR-KLU in vitro and in vivo.

## Graphical abstract

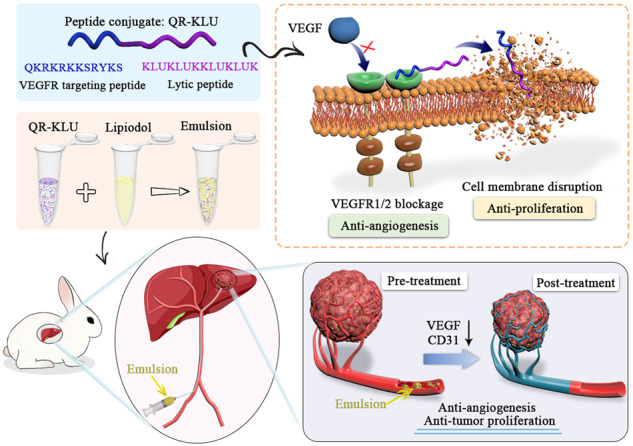

## Introduction

Hepatocellular carcinoma (HCC), the fifth most common cancer and the second leading cause of death from cancer worldwide [1], is characterized by a broad clinical and biological heterogeneity and is highly resistant to traditional therapy [2]. Unlike the surrounding hepatic parenchyma, liver tumor is preferentially vascularized by the hepatic artery rather than the portal vein. This difference in vascularisation makes it possible to selectively deliver drugs, embolic agents specifically to target tumor-feeding artery under the guidance of medical imaging equipment [3, 4]. Transcatheter arterial chemoembolization (TACE) has been confirmed the efficacy to prolong the survival of patients with HCC and recommended as the first-line treatment for unresectable HCC [1].

However, when TACE induces tumor necrosis, it can also aggravate hypoxia at the tumor site, which leads to upregulation of hypoxia-inducible factor 1α (HIF-1α) and vascular endothelial growth factor (VEGF), thus resulting in tumor-associated angiogenesis, tumor recurrence and metastasis [5, 6]. Therefore, anti-angiogenic therapy has attracted much attention after TACE procedure in liver tumors [7]. Currently, numerous therapeutic agents that target angiogenesis by blocking the VEGF signaling pathway have been developed, such as sorafenib, regorafenib, and others [8]. However, due to the lack of tumor specificity, oral-systemic administration of these anti-angiogenesis agents often lead to high toxicity and severe side effects [9]. Some researchers then proved that local administration through transcatheter arterial embolization (TAE) not only inhibited tumor angiogenesis but also reduced the side effects of these anti- angiogenesis agents in animal models [10–12]. Thus, many methods have emerged to improve the therapeutic effect of TACE such as the application of different inhibitors to disrupt VEGF or HIF-1α signal pathways, or combination with novel nanomaterials to change the hypoxia environment [11–14]. Despite these discoveries, the effect of anti-angiogenesis and anti-tumor is limited, and it still requires new tools or therapeutic drugs to pave the way for HCC therapy.

In recent decades, peptides have emerged as a new modality for cancer therapy [15]. The anti-cancer peptides include peptide inhibitors targeting aberrant protein in tumor, proapoptotic peptides disrupting mitochondrial membrane, and lytic peptides destroying cell membrane [15–17]. Among these peptides, lytic peptides are the most toxic drugs towards various cells. Due to their non-specific toxicity, lytic peptides are usually delivered to tumor tissues through targeted delivery systems such as various nanoparticles [18, 19]. Unlike chemotherapeutics, lytic peptides kill cells through disrupting cell membrane, and are often used to overcome chemo-resistance of cancer cells [20, 21]. Thus, lytic peptides could be a useful complement to chemotherapy drugs. As we mentioned above, VEGF/VEGF receptor (VEGFR) signaling is primarily responsible for tumor angiogenesis and has been considered the principal target pathway for antiangiogenic therapy. Many VEGF or VEGFR targeting peptide inhibitors have been developed for anti-angiogenesis for cancer therapy [22–24]. VEGF_125−136_ (QKRKRKKSRYKS) was first identified as an effective inhibitor to VEGFR in 2001, which was later used as efficient radiotracers for imaging VEGFR in vivo [25–27]. Due to its high affinity for VEGFR, peptide VEGF_125−136_ can be used as a favorable VEGFR ligand conjugated with other toxic drugs to kill tumor cells with high expression level of VEGFR, like PDCs.

In this paper, we designed a novel PDC which was a conjugation of VEGFR targeting peptide VEGF_125−136_ and a lytic peptide. This novel peptide conjugate may not only target VEGFR expressed on endothelial cells and inhibit angiogenesis, but also potently inhibit cancer cell proliferation through destroying cell membrane. As its different mechanism from chemotherapeutics, this PDC has a good potential for chemo-resistance cancer therapy. We recognized this peptide could be a potential drug candidate delivered through TAE for HCC therapy. As we know, there has not been a peptide inhibitor used in combination with TACE for HCC therapy. So we developed a VX2 rabbit tumor model and applied this peptide conjugate to TAE for liver cancer therapy, in which this peptide demonstrated better in vivo anti-tumor and anti-angiogenesis effect than conventional TACE. This work may provide an alternative option in combination with TACE for HCC therapy in the future.

## Results

### Design of VEGFR targeting peptide conjugate

**Fig. 1:**
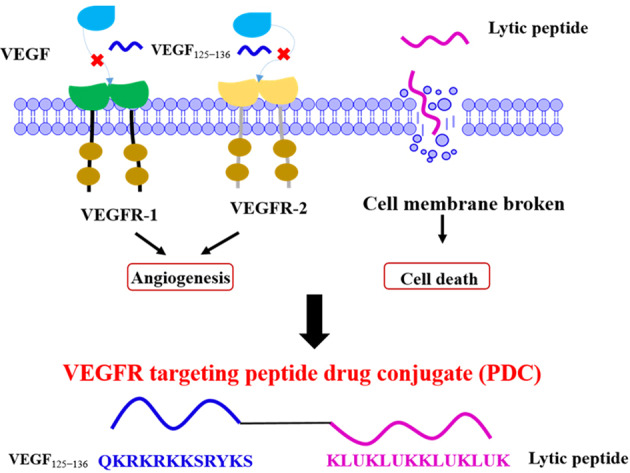
The design of QR-KLU peptide conjugate. A lytic peptide (KLU) was conjugated with VEGFR peptide ligand (QR), called QR-KLU.

Anti-angiogenesis in tumor tissue has become a key strategy in the treatment of TACE. Besides small molecules, many peptide ligands also showed good binding affinity for VEGFR and have been widely used for anti-angiogenesis in tumor or radiotracers for malignant cancers [22–24]. In this paper, we chose a reported potent VEGFR peptide inhibitor VEGF_125−136_ (QKRKRKKSRYKS, named QR) to inhibit the interaction between VEGF and VEGFR [27]. However this peptide is not potent enough to inhibit cancer proliferation. Therefore, we selected a lytic peptide (KLUKLUKKLUKLUK, named KLU) [28] to conjugate with this VEGFR peptide ligand, called QR-KLU, shown as Fig. 1. This designed peptide could not only inhibit VEGFR signal pathway, but also inhibit tumor proliferation through non-specific membrane disruption. The designed peptide conjugates could be a novel and potent agent in combination with TAE for HCC therapy.

### Cell affinity of QR-KLU peptide with endothelial cell

**Fig. 2:**
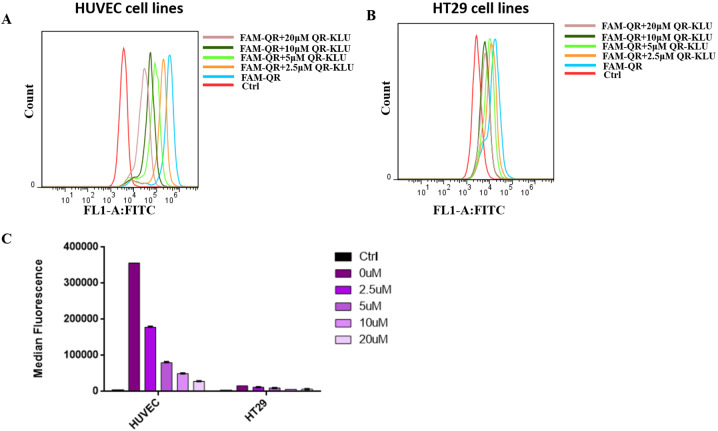
The cell affinity of QR-KLU with different cell lines. Cell penetration of peptide QR-KLU with HUVEC cells (**A**) and HT29 cells (**B**). The cells were respectively treated with PBS, 10 μM FAM-QR + 0 μM QR-KLU, 10 μM FAM-QR + 2.5 μM QR-KLU, 10 μM FAM-QR + 5 μM QR-KLU, 10 μM FAM-QR + 10 μM QR-KLU, 10 μM FAM-QR + 20 μM QR-KLU. **C** The median fluorescence of HUVEC and HT29 cells treated with different peptides as mention in Fig. 2A, B.

We then performed cell competitive binding assay to evaluate the affinity of the peptide conjugate to endothelial cell HUVEC with high expression of VEGFR [29]. FAM-labeled VEGF_125−136_ peptide QR was cultured with HUVEC cells for 4 h and then cellular penetration ability was tested by FACS. As shown in Fig. 2A, FAM labeled peptide QR showed potent cellular penetration with 0 μM peptide QR-KLU treatment, but with the increasing concentration of QR-KLU, FAM-labeled peptide QR showed decreased cell penetration ability. These results showed that QR-KLU could compete with VEGF peptide for the binding of VEGFR on cell surface under low concentration, which suggested the potent binding affinity of peptide QR-KLU to endothelial cell. To further confirm our result, we then used HT29 cells to repeat this assay as HT29 cells are reported with low expression of VEGFR [29]. We found FAM-labeled QR peptide displayed lower cell penetration in HT29 cells compared to HUVEC cells shown in Fig. 2B, C. Besides, different concentrations of QR-KLU peptide could not obviously affect cell penetration ability of FAM-labeled peptide QR. All these data suggested that QR-KLU peptide had superior affinity for endothelial cells which highly expressed VEGFR.

### Cytotoxicity of the designed peptide conjugate

**Fig. 3:**
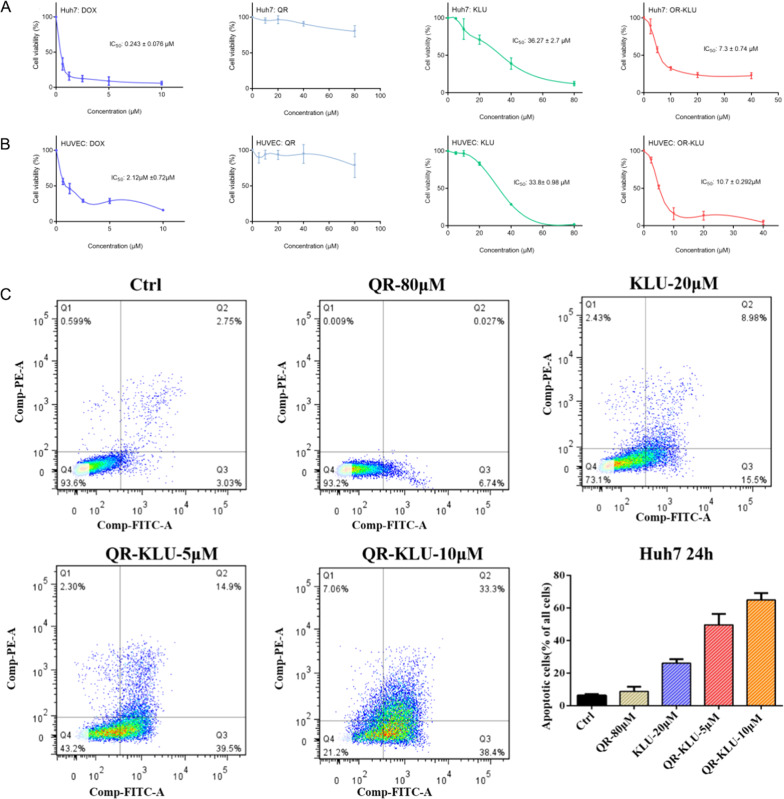
Cytotoxicity and cell apoptosis induced by different peptides. **A** Cytotoxicity of Huh7 cells treated with QR (0 μM, 10 μM, 20 μM, 40 μM, 80 μM), KLU (0 μM, 5 μM, 10 μM, 20 μM, 40 μM, 80 μM), QR-KLU(0 μM, 5 μM,10 μM, 20 μM, 40 μM) respectively for 48 h. **B** Cytotoxicity of HUVEC cells treated with different peptides the same as mentioned in Fig. 3A. **C** Cell apoptosis of Huh 7 cells induced by PBS, 80 μM QR, 20 μM KLU, 5 μM QR-KLU, 10 μM QR-KLU for 24 h.

The effect of the peptide conjugate QR-KLU on the proliferation of Huh7 and HUVEC cells was assessed by CCK8 assays. First, we investigated anti-proliferation ability of three peptides QR, KLU and QR-KLU on Huh7 and HUVEC cells with different concentrations. As shown in Fig. 3A, B, cells were treated with QR peptide, KLU peptide, QR-KLU peptide, DOX with different concentration respectively. The inhibition rate increased in a dose-dependent manner in KLU and QR-KLU groups in both cell lines. In HUVEC cells, peptide QR-KLU (IC50 10.7 ± 0.292 μM) showed more potent inhibition effect than KLU (IC50 33.8 ± 0.98 μM), and in Huh7 cells, QR-KLU (IC50 7.3 ± 0.74 μM) also showed more potent anti-tumor effect than KLU (IC50 36.27 ± 2.7 μM). Meanwhile, peptide QR showed negligible toxicity even under 80 μM. As expect, DOX showed the most potent cellular toxicity in both cell lines (IC50 0.243 ± 0.076 μM in Huh7 cells and IC50 2.12 μM ± 0.72 μM in HUVEC cells). All these data demonstrated that QR-KLU held certain cytotoxicity in vitro. We then used wound healing assay to determine whether QR-KLU could influence the cell migration of HUVEC cells (Supplementary Fig. S1). The cell proliferation or migration of HUVEC was not affected by PBS or 80 μM QR. Meanwhile, when HUVEC was treated with 20 μM KLU or 10 μM QR-KLU, the wound epithelial gap was unchanged or even bigger, indicating these two peptides could inhibit cell migration of HUVEC. All these results demonstrated that our designed peptide conjugate QR-KLU could significantly inhibit cancer cell or endothelial cell proliferation.

### Effect of QR-KLU on the cell apoptosis of Huh7 and HUVEC cells

The improved proapoptotic activity of QR-KLU was further confirmed by Annexin V-PI staining through FACS. Viable cells, early apoptotic cells, necrotic cells, late apoptotic cells were represented by Q4, Q3, Q2 and Q1 respectively. Peptide QR-KLU displayed a significant proapoptotic effect under 10 μM with apoptosis rate over 60% in Huh 7 cells and over 40% in HUVEC cells shown in Fig. 3C and Supplementary Fig. S2. Besides, QR-KLU induced cell apoptosis in a dose-dependent manner. However, peptide QR didn’t show obvious proapoptotic effect at 80 μM in both Huh 7 cells and HUVEC cells. The percentage of apoptotic cells in KLU peptide at 20 μM were about 25% in both Huh7 cells and HUVEC cells. In addition, all these peptides did not cause severe necrosis.

### Tumor growth

**Fig. 4:**
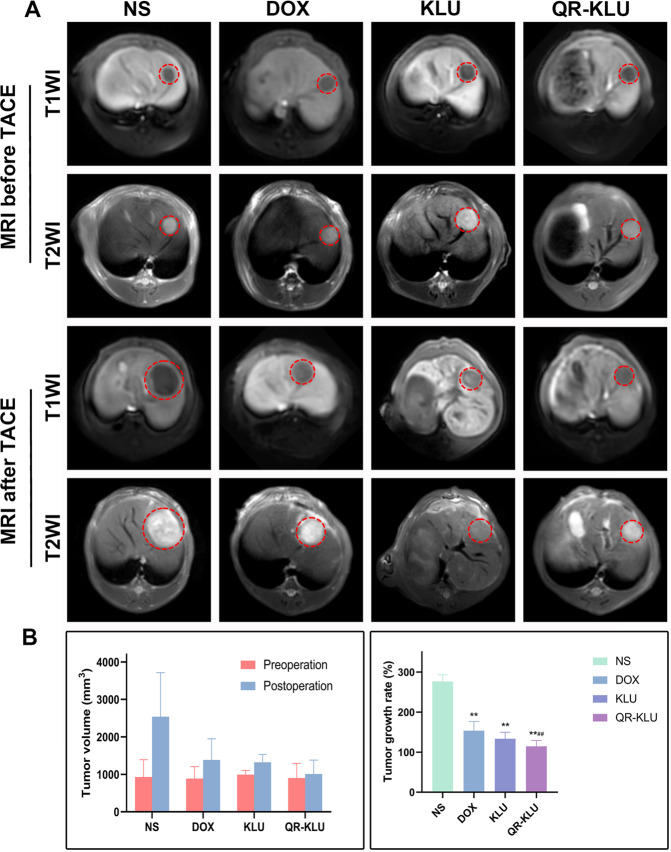
Peptide QR-KLU suppressed the tumor growth. **A** T1WI and T2WI MR images show the liver tumors in NS, DOX, KLU and QR-KLU groups Before and 7 days after TACE treatment. **B** The mean tumor volumes and tumor growth rates were calculated based on MR images in NS, DOX, KLU and QR-KLU groups. ^**^P < 0.01 versus NS group, ^##^*P* < 0.01 versus DOX group.

One day before TAE procedure, the MR images showed that the VX2 rabbit tumor models were established successfully, and the mean tumor volumes in NS, DOX, KLU and QR-KLU groups were 930.7 ± 461.1, 889.0 ± 317.7, 991.8 ± 114.2, and 899.7 ± 394.0 mm^3^, respectively shown in Fig. 4A. And at 7 days after TAE procedure, the mean tumor volumes in the four groups were 2540.5 ± 1173.8, 1382.9 ± 563.7, 1321.4 ± 210.4, and 1008.5 ± 370.7 mm^3^, respectively. The tumor volume in NS group increased significantly, with an average growth rate of 276.4%, but the tumor growth rates of DOX, KLU and QR-KLU groups were significantly lower than that of the NS group (*P* values < 0.001) shown in Fig. 4B. Notably, the mean tumor growth rate of QR-KLU group was significantly lower than the other three groups (*P* values < 0.01), but no significant difference was found between the KLU group and the DOX group (*P* > 0.05).

### Tumor necrosis, apoptosis and proliferation

**Fig. 5:**
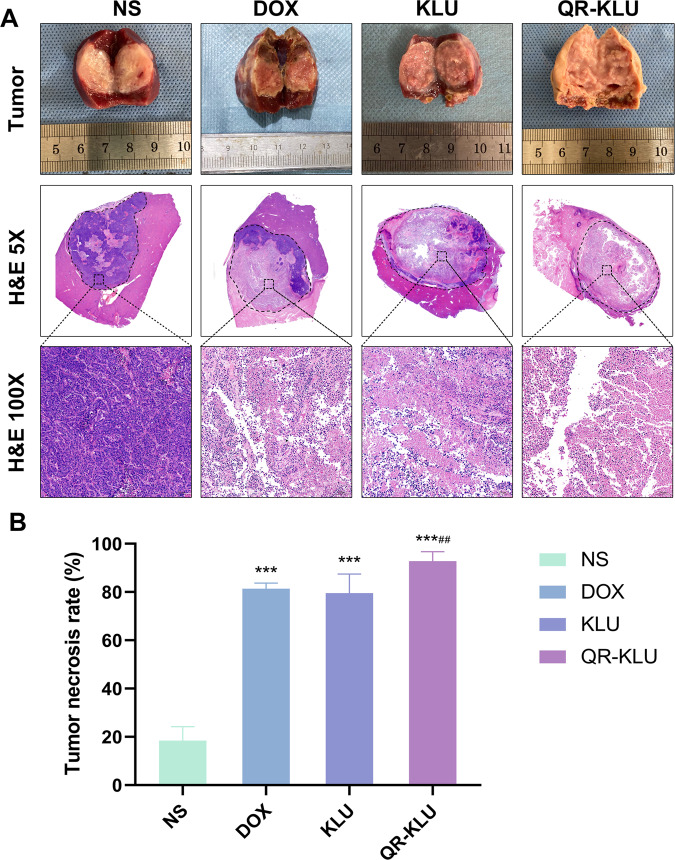
Peptide QR-KLU promotes tumor necrosis. **A** Representative pictures of the liver tumors and haematoxylin and eosin (H&E) staining of tumor tissue at 7 days after TACE treatment in NS, DOX, KLU and QR-KLU groups. **B** Tumor necrosis rates in NS, DOX, KLU and QR-KLU groups. ***P < 0.001 versus NS group, ^##^*P* < 0.01 versus DOX group.

Seven days after treatment, significant necrosis was observed in the DOX, KLU and QR-KLU groups from the gross specimens of tumors, but in NS group, the necrosis was not significant. Necrosis was measured via H&E staining, the mean tumor necrosis rates in NS, DOX, KLU and QR-KLU were 18.5% ± 5.7%, 81.4% ± 2.3, 79.5% ± 8.0% and 92.8% ± 3.9%, respectively shown in Fig. 5A. The mean necrosis rate of QR-KLU group was significantly higher than the other three groups shown in Fig. 5B (*P* values < 0.01).

**Fig. 6:**
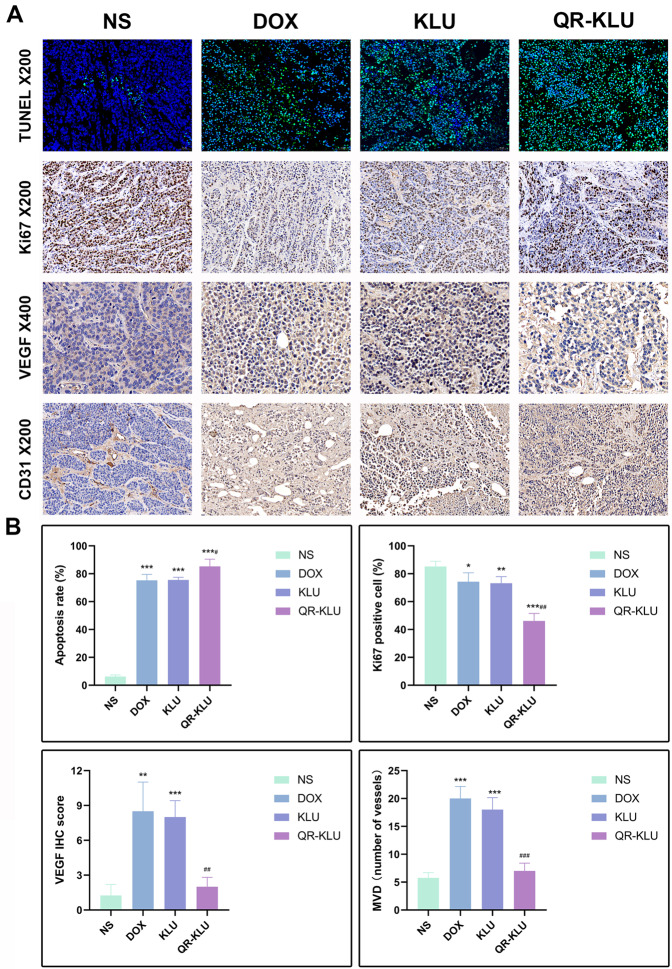
Peptide QR-KLU promotes tumor cell apoptosis, inhibits tumor cell proliferation and the expression of VEGF and CD31. **A** Representative images of TUNEL assay, Ki67, VEGF and CD31 expression detected by immunohistochemistry (IHC) staining in the four groups. **B** Comparisons of the tumor apoptosis rate, Ki67 positive cell, VEGF IHC score and microvessel density (MVD) in the four groups. ***P < 0.001 versus NS group, **P < 0.01 versus NS group, ^###^*P* < 0.001 versus DOX group, ^##^*P* < 0.01 versus DOX group, ^#^*P* < 0.05 versus DOX group.

TUNEL assay and Ki67 staining were used to evaluate tumor apoptosis and proliferation, respectively. As shown in Fig. 6, the mean proportion of apoptotic cells in DOX, KLU and QR-KLU groups was significantly higher than that in NS group (*P* values < 0.001), and the proportion was highest in QR-KLU group (*P* < 0.01). In addition, the mean proportion of proliferating cells in DOX, KLU and QR-KLU groups were significantly lower than that in NS group (*P* values < 0.001), and the proportion was lowest in QR-KLU group (*P* < 0.01).

### Angiogenesis

Compared with the NS group, after the embolization of liver tumor using lipiodol-doxorubicin emulsion or lipiodol-KLU emulsion, the VEGF IHC score and MVD increased significantly (*P* values < 0.001), this is suggestive of the increase of tumor-associated neovascularization. However, the use of QR-KLU significantly reduced the expression of CD31 and VEGF in the hypoxic tumor tissue, which suggested that QR-KLU could significantly inhibit tumor neovascularization and reduce the MVD in the tumor shown in Fig. 6.

### Therapeutic safety

In this study, we also assessed the toxicity of QR-KLU in rabbits. The mean body weight of rabbits in each group showed a trend of increasing first and then decreasing, but the rabbits in NS group lost significantly more body weight than the other groups (Supplementary Fig. S3). the gross specimens and H&E staining of hearts, lungs, kidneys and spleens showed no apparent histopathological abnormality (tissue damage and/or inflammation) (Supplementary Figs. S4 and S5).

Subsequently, we analyzed the biochemical indicators of liver and renal function in tumor-bearing rabbits before and at 1, 3, and 7 days after treatment. As shown in Supplementary Fig. S6, no significant difference was found among the four groups in the liver and renal function indicators (ALT, AST, TBIL and CREA). This is suggestive of no significant liver or renal toxicity of QR-KLU and KLU.

## Discussion

Transcatheter arterial chemoembolization (TACE) is considered as an important interventional therapeutic approach for hepatocellular carcinoma (HCC) [30], and it is a minimally invasive procedure to deliver chemotherapeutic drugs and embolic materials to liver tumor through the feeding artery. Besides, TACE could avoid giving a high dose of drug via systemic drug administration, minimize off-target side effects and amplify anti-tumor activity [31–33]. However, the inhibiting effect of tumor progression after TACE is usually limited, most likely because hypoxic stress generated by embolization and the up-regulation of vascular endothelial growth factor (VEGF), eventually leading to neoangiogenesis and tumor recurrence [34–37]. Therefore, the application of anti-angiogenic agents could theoretically inhibit post-TACE neovascularization and decrease cancer relapse. Nowadays, multiple clinical trials have confirmed the good effect of the combination of TACE and oral anti-angiogenic agentsfor HCC therapy [38, 39]. However, the side effect of oral anti-angiogenic agents are also obvious in cancer patients. The local delivery of antiangiogenic agents via TAE might benefit patients with good therapeutic effect and safety. And many researches have proved that local delivery of antiangiogenic agents via TAE can inhibit in vivo tumor angiogenesis and tumor progression, which promote the development of therapeutic approaches for HCC [10–12].

Peptides have been widely used for cancer therapy with potent cellular toxicity. Due to the quite different anti-cancer mechanism from chemotherapeutics, lytic peptides have attracted much attention for chemo-resistant cancer therapy via local delivery systems [40, 41]. Besides, many lytic peptides were hybrided with target ligands to improve their selectivity for targeted cancer cells [42, 43]. Despite these lytic peptides are widely used for anti-cancer therapy, they have not been used in combination with TAE for HCC therapy. In this paper, we designed a dual functional peptide inhibitor (QR-KLU) with a conjugation of VEGFR targeting peptide VEGF_125−136_ and a reported lytic peptide. We evaluated the antineoplastic efficacy of peptide QR-KLU in vitro and in vivo. Peptide QR-KLU markedly inhibited the growth of Huh7 cells and HUVEC cells, and the inhibition effect was more potent than single lytic peptides (KLU). And the wound-healing assay showed that QR-KLU could effectively inhibit the HUVEC cell migration. The above results indicate that our designed peptide conjugates QR-KLU could significantly inhibit liver cell or endothelial cell proliferation. In addition, compared with single lytic peptides, peptide QR-KLU significantly promoted the apoptosis of Huh7 cells and HUVEC cells by flow cytometric analyses. In in vivo experiments, peptide QR-KLU also showed stronger anti-tumor activity in VX2 rabbit tumor model than the DOX group and more obvious tumor necrosis and apoptosis, which the necrosis rate of liver tumors reached 93%. We also found that QR-KLU group could decrease the expression of VEGF and CD31 further, confirming peptide QR-KLU could inhibit tumor angiogenesis. In addition, we studied the safety of peptide QR-KLU through H&E staining of hearts, lungs, kidneys and spleens, which showed no apparent tissue injury. And the biochemical indicators of liver and renal function did not show a significant difference between peptide QR-KLU group and NS group. Therefore, we believed that the use of peptide QR-KLU in combination with TAE is safe and feasible for HCC therapy.

In conclusion, Our research provides a new option for the TACE treatment of HCC. Compared with the conventional chemotherapeutic agents, peptide QR-KLU exhibits better anti-tumor activity and anti-angiogenesis effect, and has a favorable safety profile. Thus, peptide QR-KLU holds a promising application prospect in the TACE treatment of liver cancer and deserves further study.

## Materials and methods

### Peptide synthesis

The peptide synthesis was based on the solid phase peptide synthesis (SPPS) as we reported before [44]. Details of the synthesis of peptide inhibitors and their structure analysis were given in the Supplementary Fig. S7, and the high performance liquid chromatography (HPLC) and mass spectrometry (MS) data of different peptide were shown in Supplementary Figs. S8–S11.

### Tumor cell lines and culture

Human liver cancer cell lines, Huh7 cells, human umbilical vein endothelial cells (HUVEC) and human colon cancer cell HT29 were cultured in dulbecco’s modified eagle medium (DMEM) with 10% (v/v) fetal bovine serum (FBS) and penicillin/streptomycin (0.1 mg/mL). All reagents were purchased from Gibco. All these cells were were routinely tested for mycoplasma contamination and tested negative and maintained in a humidified incubator containing 5% CO_2_ at 37 °C.

### Cell viability assay

Cell viability for different cell lines were measured by cell counting kit-8 (CCK8) assays. The cells were incubated on a 96-well plate for 24 h in growth medium prior to drug treatment. Then the medium was removed followed by adding different concentrations of peptides in medium with 5% FBS (v/v) for 48 h’ incubation, replaced by 10% FBS medium for another 24 h, if necessary, as mentioned in other studies [44, 45]. Then CCK8 (10 μL) was added and the cells were incubated for 1 h at 37 °C with 5% CO_2_. Absorbance was measured with a microplate reader (Bio-Rad) at a wavelength of 450 nm.

### Cell affinity assay

The affinity ability of peptide QR-KLU to endothelial cells was determined by fluorescence-activated cell sorting (FACS) analysis. Briefly, the HUVEC cells were incubated on a 24-well plate for 24 h in growth medium. Then peptide QR-KLU in medium with different concentrations (0 μM, 2.5 μM, 5 μM, 10 μM, 20 μM) were respectively incubated with HUVEC cells for 30 min at 37 °C. Next, carboxy-fluorescein (FAM)-labelled-QR peptide (10 μM) were added to each well for 4 h at 37 °C. Fluorescent labeling methods have been described in previously published articles [23, 46]. After incubation, the cells were harvested with 0.25% trypsin/ethylene diamine tetraacetic acid (EDTA) and washed twice with PBS. The cell pellet was then resuspended in 0.5 mL of PBS. FACS was performed and the results were analyzed using FlowJo.

### Cell apoptosis assay

The apoptosis assay was performed according to the manufacturer’s instructions using an Annexin V: Fluorescein Isothiocyanate (FITC) Apoptosis Detection Kit I (BD Pharmingen™). Briefly, Huh 7 or HUVEC cells were seeded in a 12-well plate and allowed to grow for 24 h in medium with 10% FBS. Then, the cells were treated with the different peptides in medium with 5% FBS for 24 h’ incubation. The cells were harvested with 0.25% trypsin/EDTA, washed twice with PBS and suspended in 1 × binding buffer. The suspended cells were treated with FITC-labeled Annexin V and propidium iodide (PI) as the protocol indicated and then analyzed by flow cytometry to determine the apoptotic cells. The cells with positive fluorescence intensity signals for both FITC and PI were used for the apoptotic cell count.

### Wound-healing assay of HUVEC cells

Wound-healing assay was performed according to the protocols reported by Liang et al. [47]. Briefly, HUVEC cells were plated into 12-well plate and grown to create a confluent monolayer. The monolayer was grazed in a straight line to create a “scratch” with a pipette tip. Markings were created close to the scratch to be used as reference points. Then cells were treated with different peptides for 24 h at 37 °C. Images were collected with a Leica DFC480 camera on a phase-contrast microscope equipped with a 10 × objective at room temperature. The free area of the wound was measured by image software.

### VX2 rabbit tumor model

New Zealand white rabbits (2.5–3 kg) were purchased from the Laboratory Animal Center of Tongji Medical College of Huazhong University of Science and Technology (Wuhan, China). All the animal experimental procedures were performed in accordance with the National Institutes of Health Guidelines for the Care and Use of Laboratory Animals and the protocol was approved by the Ethics Committee of Tongji Medical College, Huazhong University of Science and Technology. Blinding was not possible in most animal experiments.

The rabbit VX2 liver tumor model was established according to the method reported by Qian K et al. [48]. In brief, after the rabbit was fully anesthetized, the abdominal cavity was cut open along the abdominal midline below the xiphoid, then a prepared VX2 tumor particle (volume: 1 mm^3^) was embedded in the left liver lobe. The growth of VX2 tumor in rabbit liver lobe was evaluated using a 3.0-T magnetic resonance (MR) system (Ingenia CX, Philips Healthcare, Best, the Netherlands) with an eight-channel phased-array small flexible coil (ds SmallExtr 8ch, Invivo Corporation, Avenue Gainesville, USA). The acquisition parameters of the sequence are as follows: T2WI, TR 2750 ms, TE 65 ms, the field of view 120 mm × 120 mm, Slice thickness 3 mm in the transverse plane, matrix 152 × 152. The multi-vane technique was used to reduce artifacts and improve image quality, acquisition time 3 min 202 s; T1WI 3D Fast field echo sequence with the multi-vane technique, TR 3.3 ms, TE 1.38 ms, the field of view 120 mm × 120 mm, Slice thickness 3 mm in transverse plane, matrix 152 × 152, acquisition time 3 min 20 s.

### Groups and TACE procedure

Twenty-four VX2 tumor-burdened rabbits were evenly divided into four groups. After fasting for 12 h, the rabbits were anesthetized by intravenous injection of pentobarbital sodium solution (2 wt.%, 30 mg/kg). The rabbit’s right femoral artery was dissected and a 4-F Cobra catheter (Cook, Inc., Bloomington, Indiana) was inserted into the celiac artery, hepatic arteriography was then performed under the guidance of digital subtraction angiography (DSA), and a 2.7-F coaxial microcatheter (Terumo, Tokyo, Japan) was super-selectively inserted into the tumor donor branch subsequently. The emulsion of drugs and lipiodol were slowly injected into the tumor-feeding artery through the above microcatheter at a speed of 0.5 mL/min. Each group was injected as follows: negative saline (NS) group (0.5 mL saline); DOX group (0.5 mL lipiodol-doxorubicin emulsion); KLU group (0.5 mL lipiodol-KLU emulsion); QR-KLU group (0.5 mL lipiodol-[QR-KLU] emulsion). The dose of DOX (molecular weight [MW]: 543.52 g/mol), KLU (MW: 1621.18 g/mol) and QR-KLU (MW: 3309.25 g/mol) were 2 mg/kg, 4 mg/kg and 4 mg/kg, respectively. After obtaining all DSA images, the sheaths and catheters were removed. The femoral artery was ligated, the muscle, subcutaneous tissue and skin were sutured, and penicillin (20,000 IU/day) was injected intramuscularly for 3 days to prevent infection (Supplementary Fig. S12).

Seven days after TACE procedure, tumor size was evaluated again by MR scan and the tumor volume was calculated by formula: V = a * b^2^/2 (a, long diameter; b, short diameter). And tumor growth rate was calculated by formula: V_7_/V_0_ × 100% (V_0_ and V_7_ represent the tumor volume at baseline and 7 days after treatment, respectively). Finally, the VX2 rabbits were sacrificed and the tumor tissues were harvested for biochemical analysis and histopathological examination.

### Histological analysis and immunohistochemistry (IHC)

Seven days after treatment, the liver tumors, hearts, lungs, kidneys and spleens were harvested and fixed in 4% phosphate-buffered paraformaldehyde, the specimens were embedded in paraffin blocks and cut into 5‐μm‐thick sections. Following the manufacturer’s guidelines, the sections were stained with haematoxylin and eosin (H&E) for the evaluation of organ toxicity and tumor necrosis, Ki-67 staining and transfer-mediated dUTP nick end labeling (TUNEL) technique were used to detect tumor proliferation and apoptosis, respectively.

The IHC staining procedures were performed as previously described [49]. Briefly, the sections were blocked in 3% H_2_O_2_ for 25 min and then incubated with goat serum for 15 min. Subsequently, the sections were first washed and incubated with anti-VEGF (1:200, Abcam, USA), and anti-CD31 (1:20; Abcam, USA) overnight at 4 °C, and then incubated with second antibody (Abcam, USA) at 37 °C for 50 min. Next, the sections were stained with 3, 3ʹ-diaminobenzidine (DAB, Service bio, Wuhan Saiwell Biotechnology Co., Ltd., China) for 6 min and rinsed and stained in hematoxylin for 30 s. The sections were then dehydrated in gradient alcohol and sealed in neutral resins.

VEGF expression was assessed by semiquantitative IHC based on staining intensity and density, the staining intensity was scored as 0 (negative), 1 (weak), 2 (moderate), and 3 (strong), while the staining density was scored on the basis of the percentage of positively stained cells: 0 (0%), 1 (1–25%), 2 (26–50%), 3 (51–75%), and 4 (76–100%). The total VEGF IHC score was calculated by multiplying the intensity score by the density score [50]. Microvessel density (MVD) was calculated according to the method developed by Weidner et al. [51], the CD31-stained endothelial cells or cell clusters could be considered as a microvessel, the microvessels were counted at three high magnification fields (×200 magnification) and the final result was the mean value of three fields.

### Serum biochemical analysis

Blood samples were collected before treatment and at 1, 3, 7 days after treatment. The blood samples were stood at room temperature for 30 min, and the serum was obtained by centrifugation (room temperature, 8000 rpm, 10 min) for assessment of alanine aminotransferase (ALT), aspartate aminotransferase (AST), total bilirubin (TBIL) and creatinine (CREA). The assays were performed using the commercially ready-made kits following the manufacturers’ instructions.

### Statistical analysis

Data processing and analyses were performed by using IBM SPSS statistics version 22.0 (IBM, Chicago, IL). All data were expressed as the means ± standard deviations (SDs), and differences were compared using One-Way ANOVA. *P* < 0.05 considered to be statistically significant difference. GraphPad Prism V8.0 (GraphPad Software, San Diego, CA, USA), 3D Studio Max software (Discreet Logic, Montreal, Canada) and Figdraw (Home for researchers, Hangzhou, China) were used for graphical presentations.

## Supplementary information

Supplementary figure legends

Supplementary Figure S1

Supplementary Figure S2

Supplementary Figure S3

Supplementary Figure S4

Supplementary Figure S5

Supplementary Figure S6

Supplementary Figure S7

Supplementary Figure S8

Supplementary Figure S9

Supplementary Figure S10

Supplementary Figure S11

Supplementary Figure S12

## Data Availability

The corresponding author will provide the original data used to support the findings of this study upon reasonable request.
