# Supplementary material for: A VEGFR targeting peptide-drug conjugate (PDC) suppresses tumor angiogenesis in a TACE model for hepatocellular carcinoma therapy
Source: Cell Death Discov. 2022 Oct 6;8:411. doi: 10.1038/s41420-022-01198-9 (PMC9537177; doi:10.1038/s41420-022-01198-9)
Supplement: Supplementary file 1 — Supplementary figure legends [file 41420_2022_1198_MOESM1_ESM.docx]

**Supplementary figure legends**

**Supplementary Figure S1.** Wound healing assay to detect the cell migration influenced by different peptides. HUVEC cells were respectively treated with PBS, 80μM QR, 20μM KLU, 10μM QR-KLU for 24 hours.

**Supplementary Figure S2.** Cell apoptosis of HUVEC induced by different peptides. HUVEC cells were treated with PBS, 80μM QR, 20μM KLU, 5μM QR-KLU, 10μM QR-KLU for 24 hours before detection by FACS.

**Supplementary Figure S3.** A. Work plan for *in vivo* studies. B. The body weights of all rabbits after TACE treatment in the four groups.

**Supplementary Figure S4.** Gross morphology of hearts, lungs, kidneys and spleens in the four groups.

**Supplementary Figure S5.** H&E staining of different organs at 7 days after TACE treatment in the four groups.

**Supplementary Figure S6.** Biochemical indicators of liver and renal function after TACE treatment in the four groups. (A) Alanine aminotransferase (ALT). (B) Aspartate aminotransferase (AST). (C) Total bilirubin (TBIL). (D) Creatinine (CREA).

**Supplementary Figure S7.** Peptide QR-KLU synthesis through Solid phase peptide synthesis (SPPS). Peptides were synthesized on MBHA resin (loading capacity: 0.5 mmol/g) (HECHENG S&T Ltd) via standard Fmoc-based solid-phase peptide synthesis. Firstly, the resin was swollen in DCM for 20 min, filtered, then treated with 50% (vol/vol) morpholine in NMP for 30min × 2. The resin was washed sequentially with DCM and NMP (5x). Fmoc-protected amino acids (5.0 equiv) and HATU (4.9 equiv) were dissolved in DMF, followed by DIPEA (10.0 equiv). The mixture was pre-activated for 1 min and added to the resin for 1hour with N_2_ bubbling. The resin was washed sequentially with DCM, NMP (5x) and methanol (5x) then dried under a stream of nitrogen for next step. For the acetylation of resin, the protected resin was treated with DCM/DIEA/Ac2O (8:1:0.5). For cleavage of resin, the final resin were treated with TFA/DCM/TIS/water (90:5:2.5:2.5) at room temperature for 3 h and concentrated under a stream of nitrogen. The peptide was precipitated and washed three times in cold diethyl ether and isolated by centrifugation. The dried precipitate was then dissolved in 50% (vol/vol) water/acetonitrile, purified by analytic reversed phase HPLC (Waters 600, Agilent Zorbax SB-Aq: 4.6 x 250 mm, 220 nm & 254 nm) and analyzed by LC-MS (AB SCIEX Elite QSTAR or Shimazu LC-MS 2020).

**Supplementary Figure S8.** High performance liquid chromatography (HPLC) (A) and mass spectrometry (MS) (B) data of KLU peptide.

**Supplementary Figure S9.** High performance liquid chromatography (HPLC) (A) and mass spectrometry (MS) (B) data of QR peptide.

**Supplementary Figure S10.** High performance liquid chromatography (HPLC) (A) and mass spectrometry (MS) (B) data of QR-KLU peptide.

**Supplementary Figure S11.** High performance liquid chromatography (HPLC) (A) and mass spectrometry (MS) (B) data of carboxy-fluorescein (FAM)-labelled-QR peptide peptide.

**Supplementary Figure S12.** Schematic description of TACE procedure under the guidance of digital subtraction angiography (DSA). (A) Angiography was obtained after the catheter was advanced into the celiac artery. (B) The microcatheter was super-selectively advanced into the tumor-feeding artery, and the arteriography shows hypervascular tumor staining (red circle) in the left lobe of the liver. (C) After the injection of the emulsion of peptide conjugate and lipiodol, post-embolization angiography shows the lipiodol deposition (red circle) and the complete embolization of tumor-feeding artery. CHA: common hepatic artery; GDA: gastroduodenal artery; LHA: left hepatic artery; RHA: right hepatic artery.
