# Supplementary figures and images for: A VEGFR targeting peptide-drug conjugate (PDC) suppresses tumor angiogenesis in a TACE model for hepatocellular carcinoma therapy
Source: Cell Death Discov. 2022 Oct 6;8:411. doi: 10.1038/s41420-022-01198-9 (PMC9537177; doi:10.1038/s41420-022-01198-9)

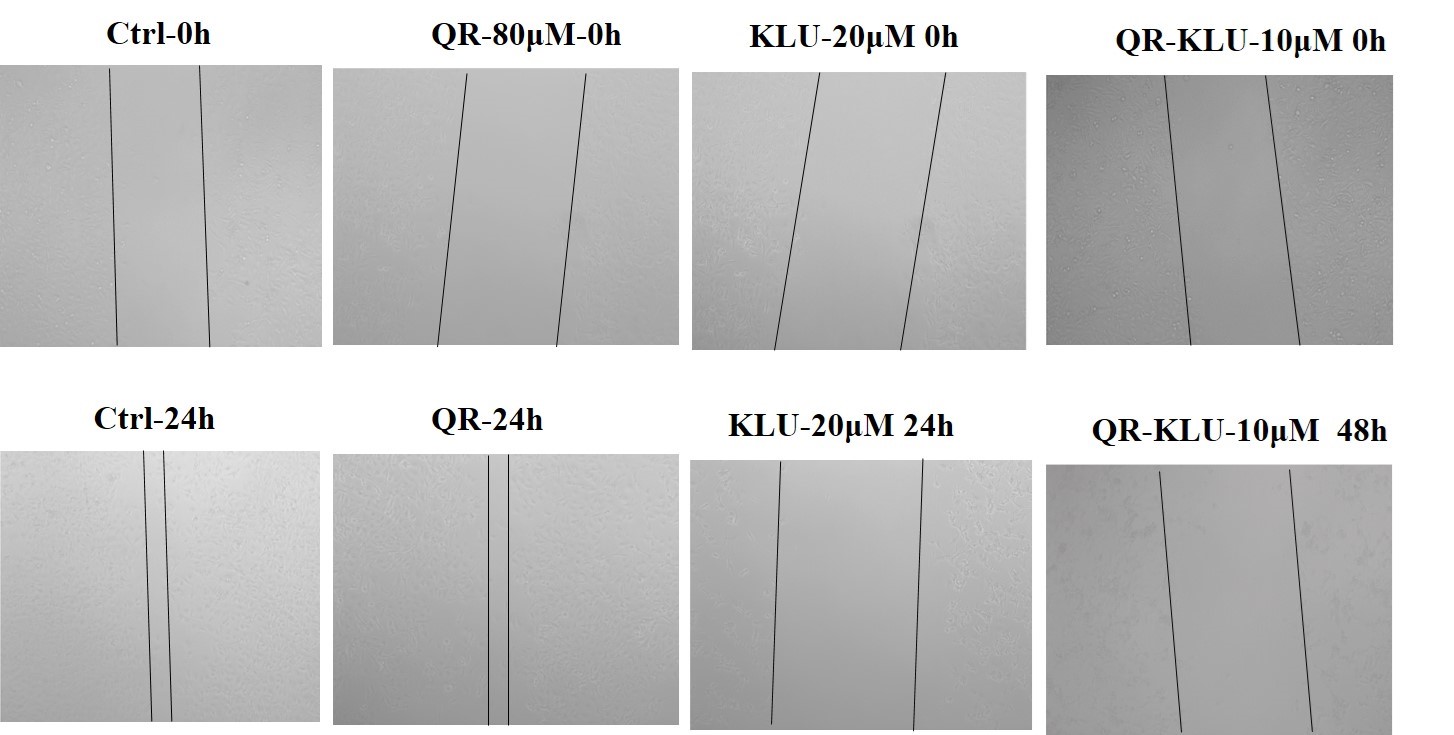

Supplement: Supplementary file 2 — Supplementary Figure S1 [file 41420_2022_1198_MOESM2_ESM.jpg]

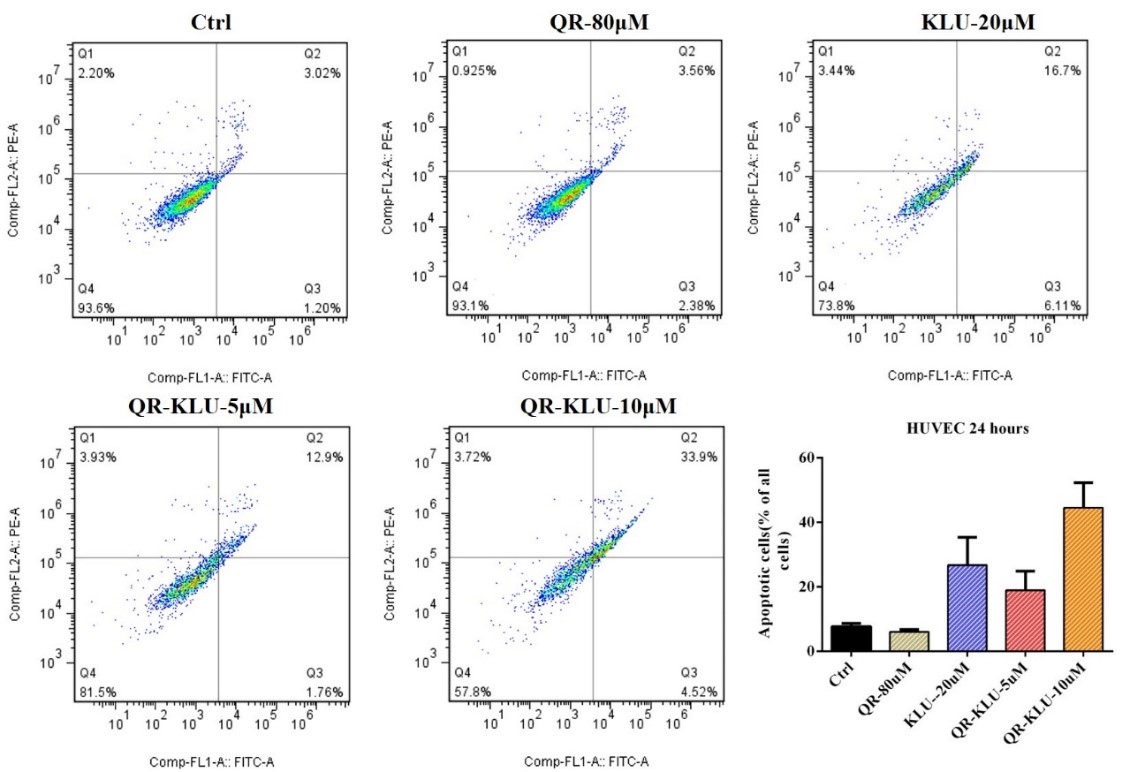

Supplement: Supplementary file 3 — Supplementary Figure S2 [file 41420_2022_1198_MOESM3_ESM.jpg]

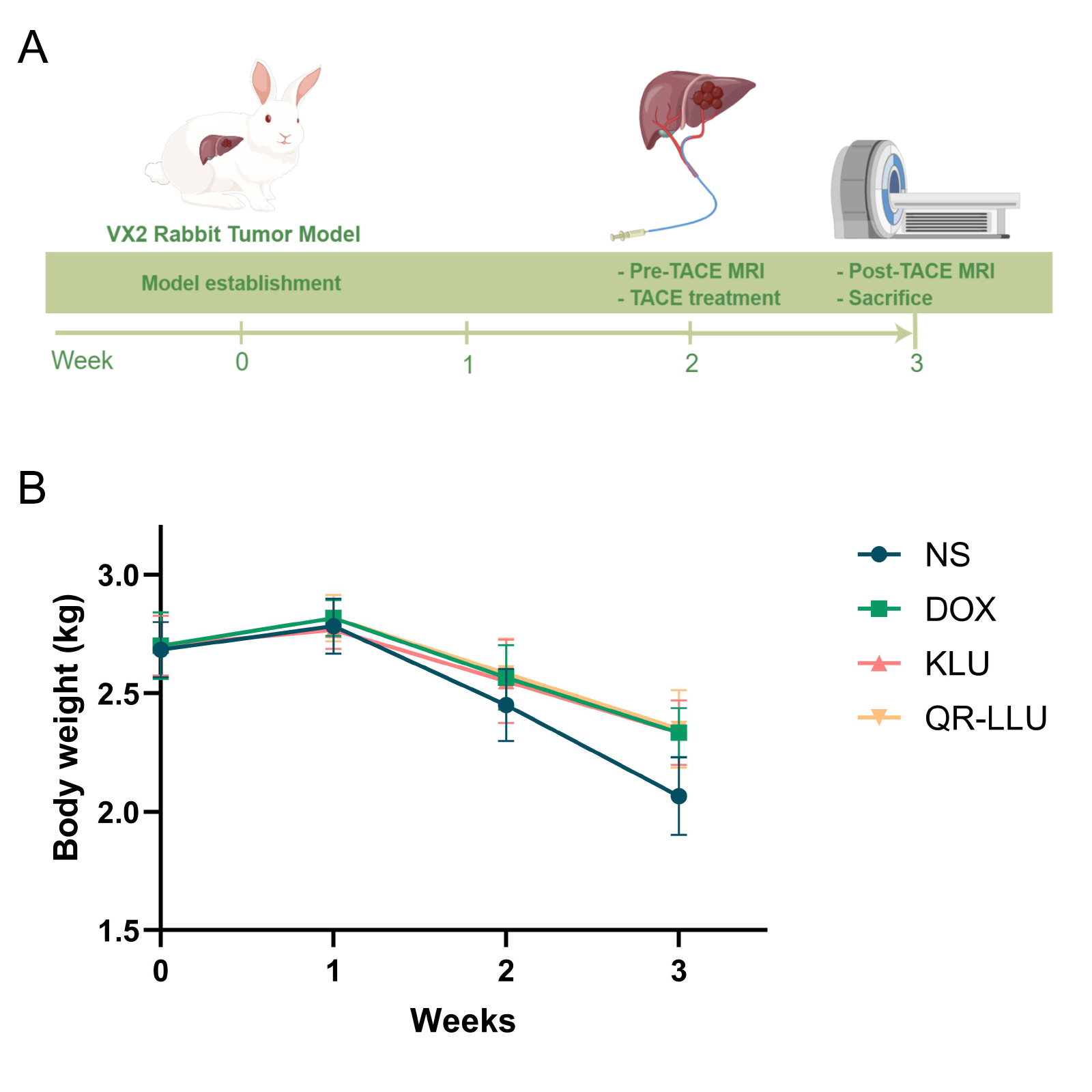

Supplement: Supplementary file 4 — Supplementary Figure S3 [file 41420_2022_1198_MOESM4_ESM.jpg]

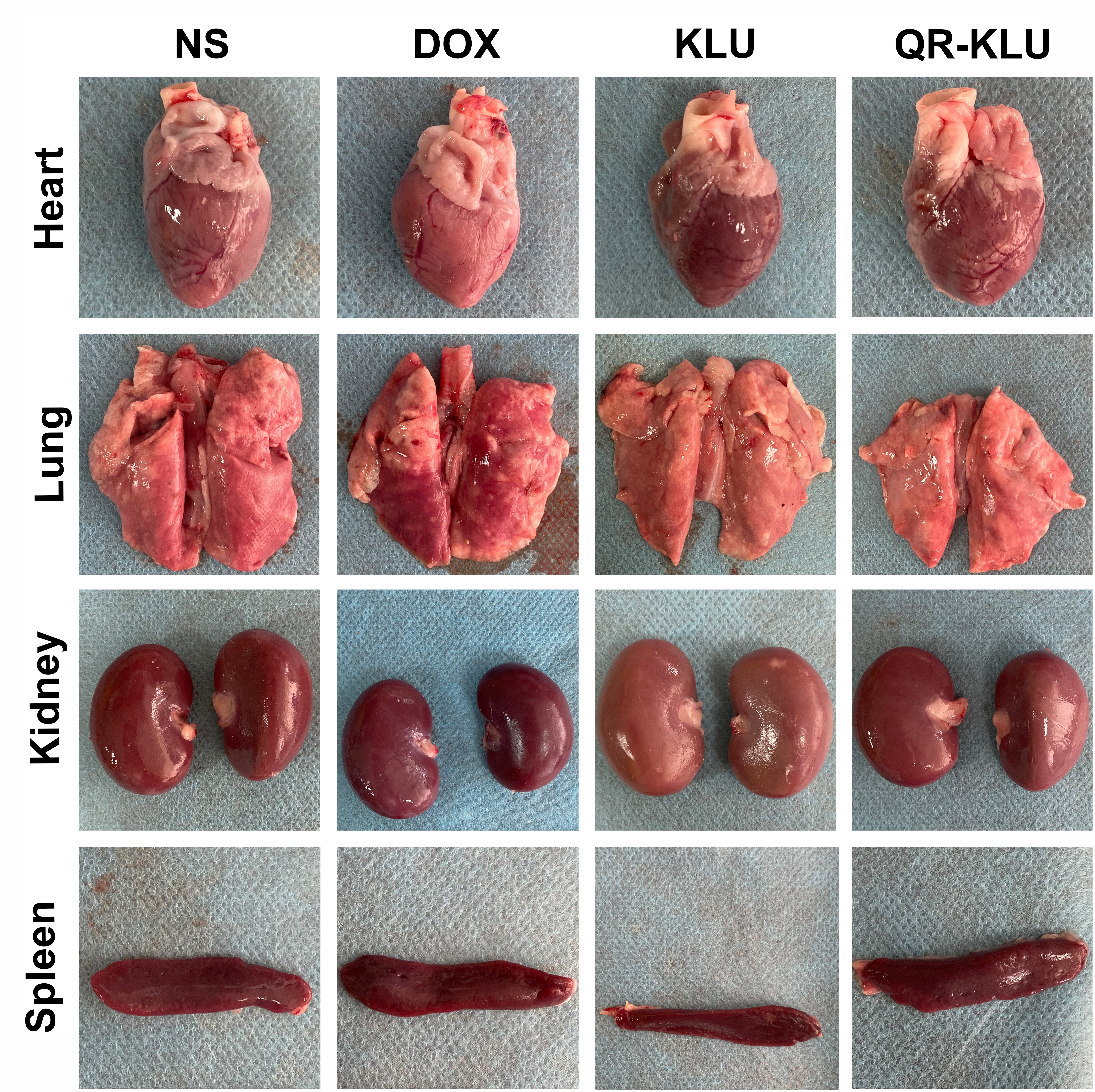

Supplement: Supplementary file 5 — Supplementary Figure S4 [file 41420_2022_1198_MOESM5_ESM.jpg]

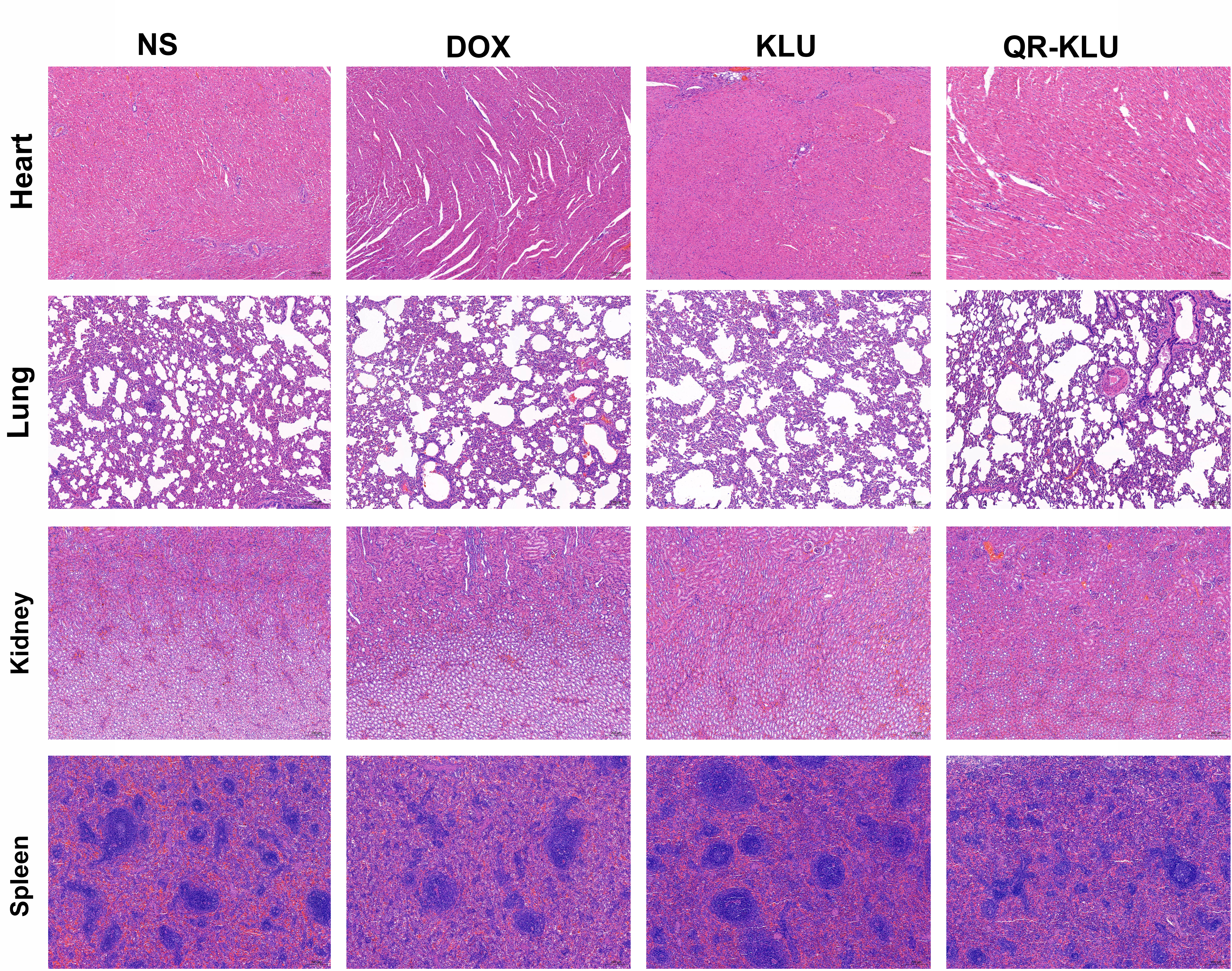

Supplement: Supplementary file 6 — Supplementary Figure S5 [file 41420_2022_1198_MOESM6_ESM.jpg]

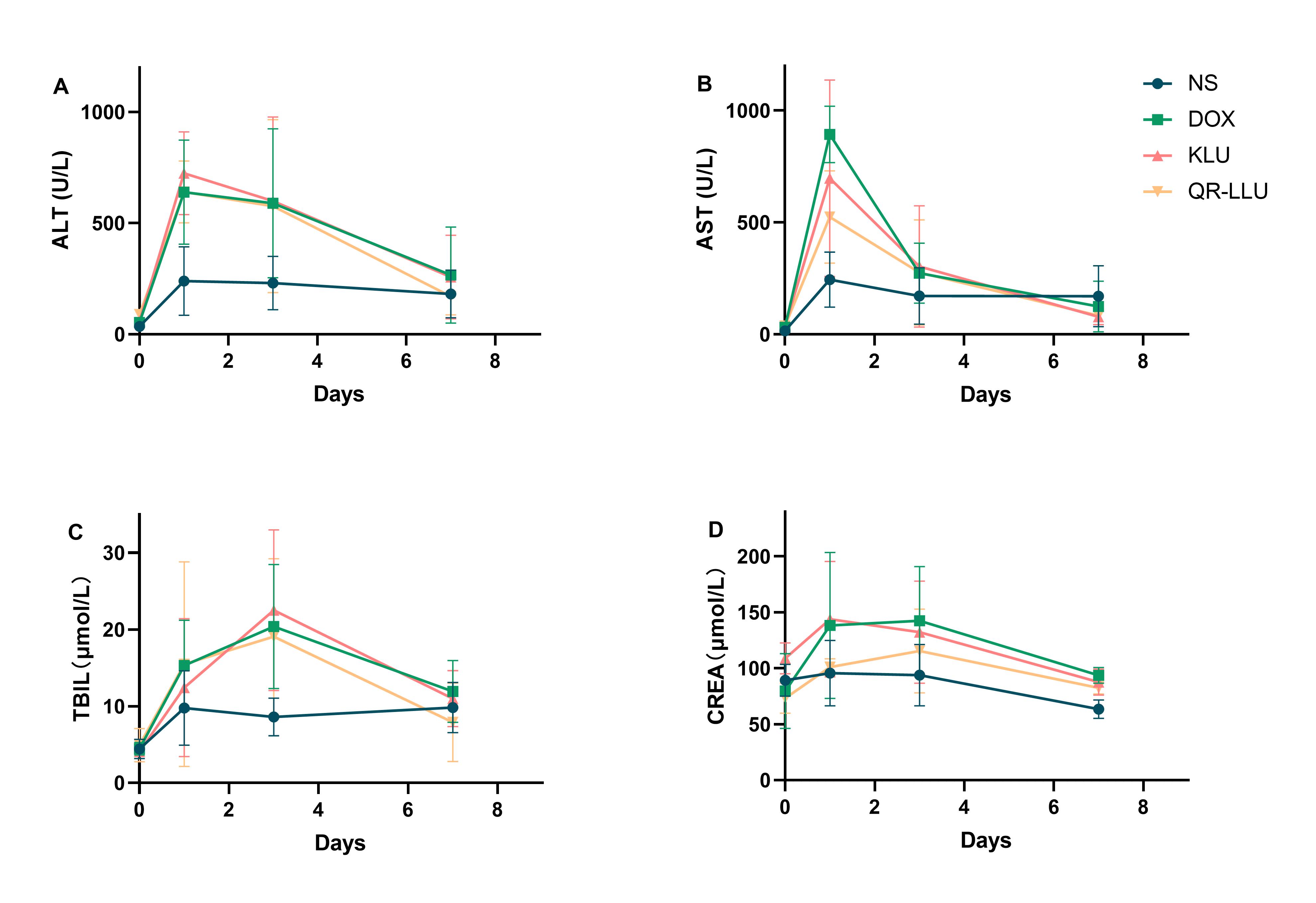

Supplement: Supplementary file 7 — Supplementary Figure S6 [file 41420_2022_1198_MOESM7_ESM.jpg]

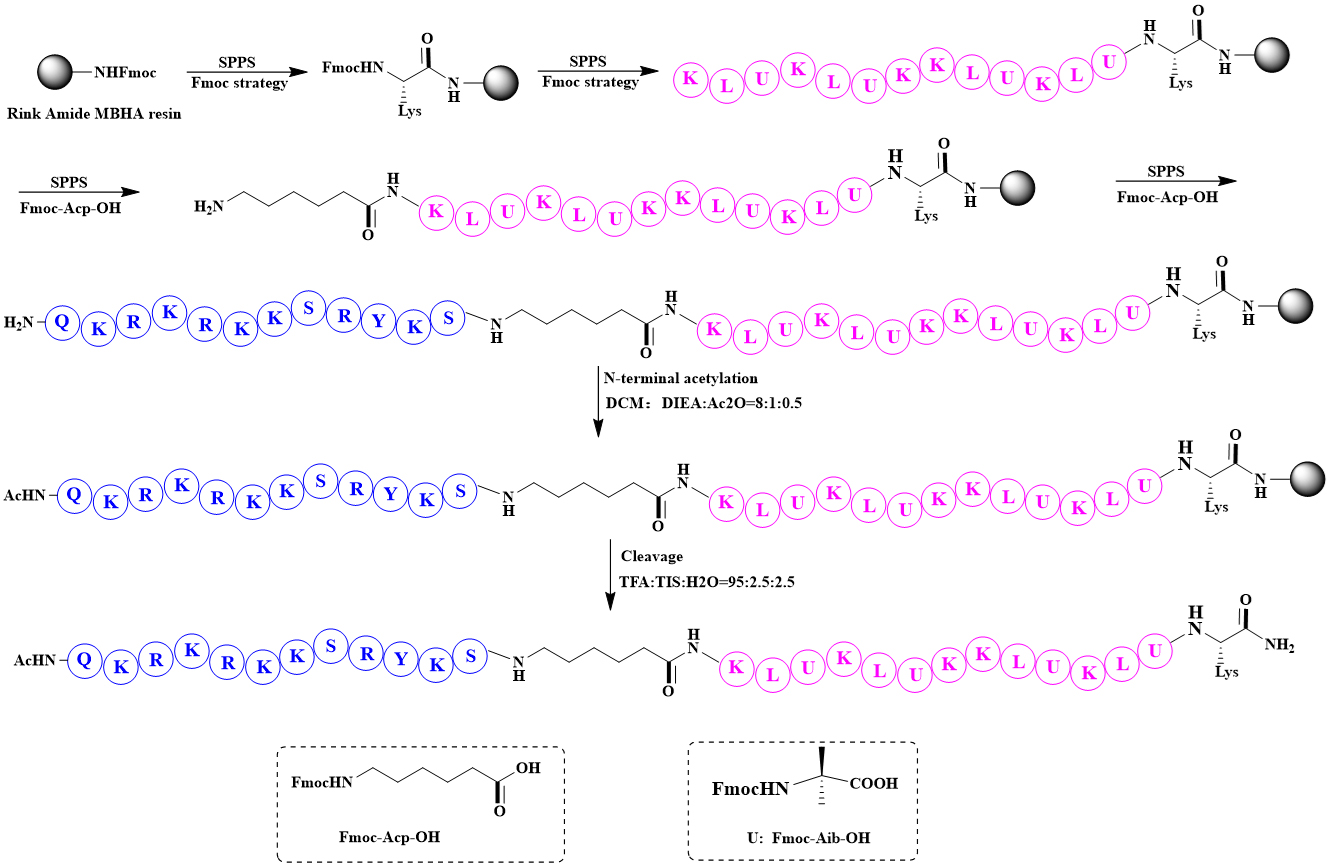

Supplement: Supplementary file 8 — Supplementary Figure S7 [file 41420_2022_1198_MOESM8_ESM.jpg]

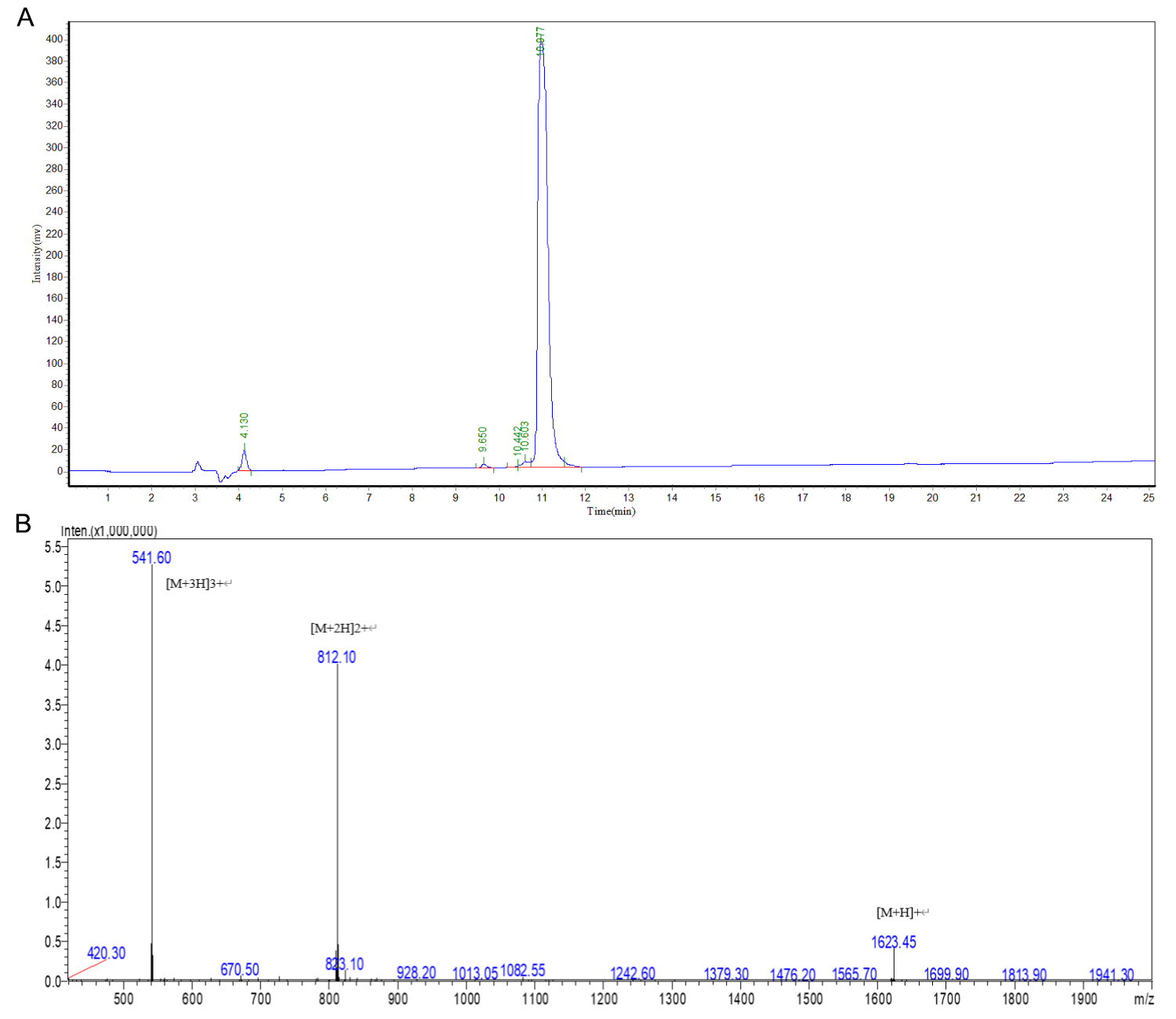

Supplement: Supplementary file 9 — Supplementary Figure S8 [file 41420_2022_1198_MOESM9_ESM.jpg]

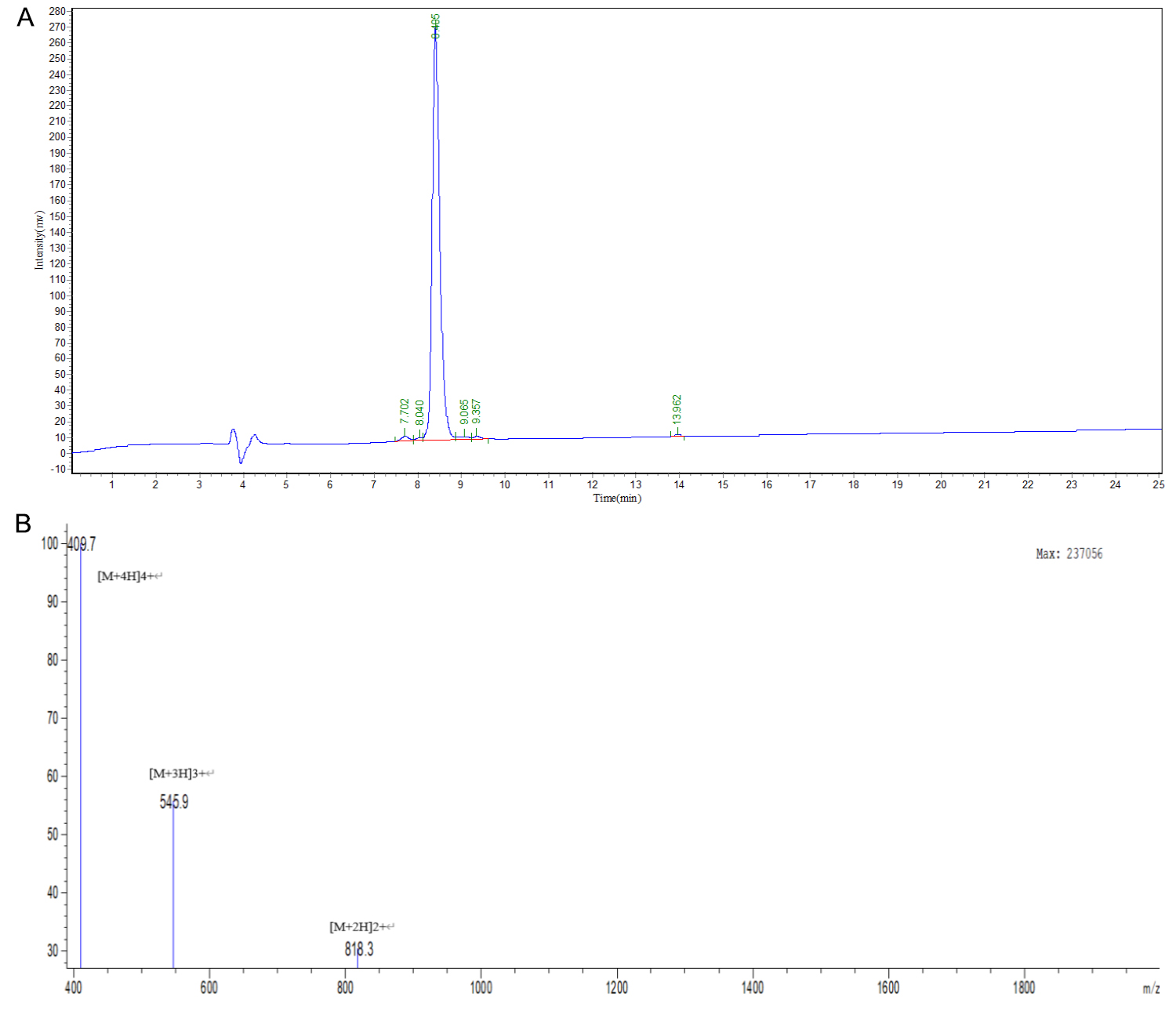

Supplement: Supplementary file 10 — Supplementary Figure S9 [file 41420_2022_1198_MOESM10_ESM.jpg]

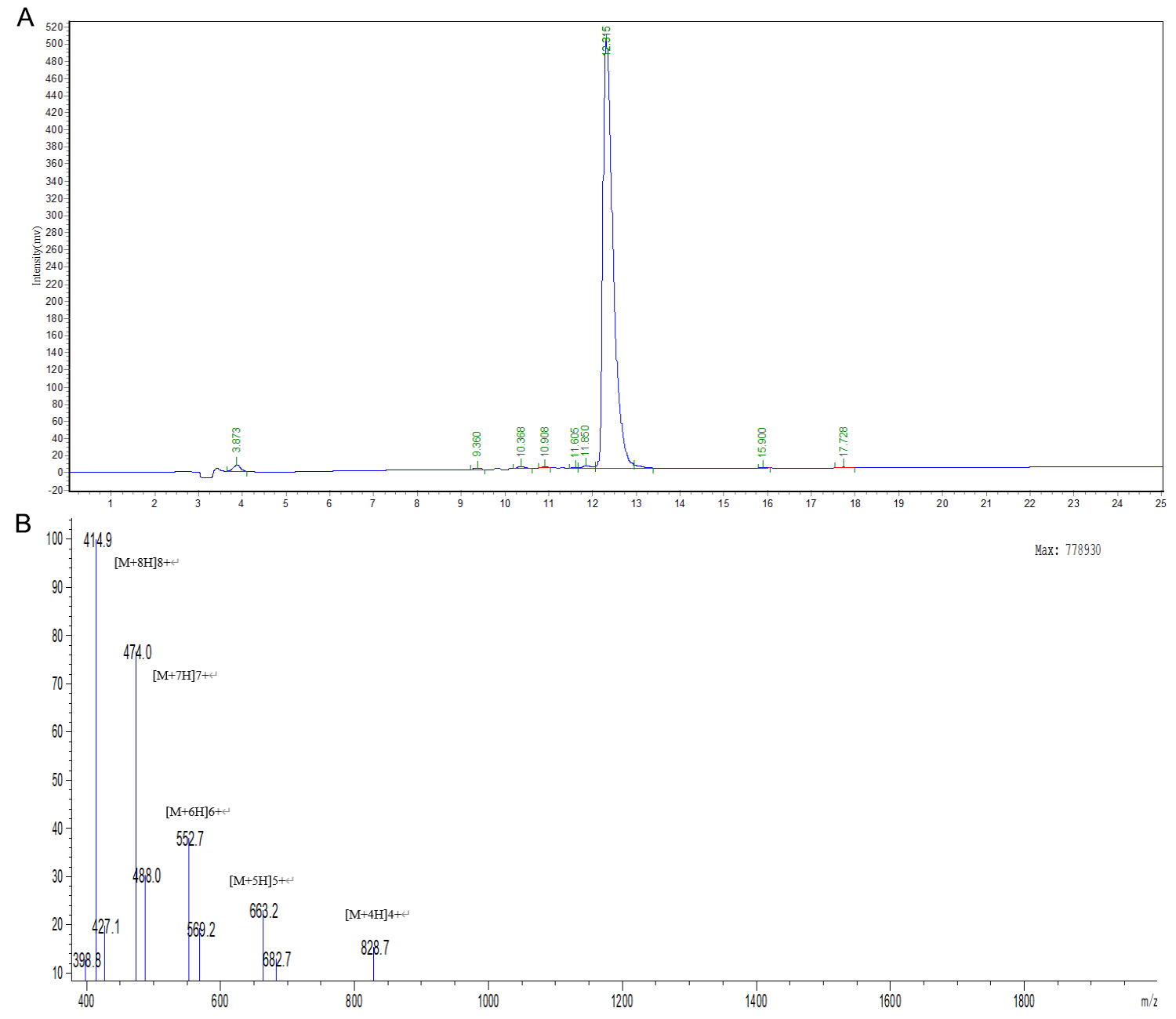

Supplement: Supplementary file 11 — Supplementary Figure S10 [file 41420_2022_1198_MOESM11_ESM.jpg]

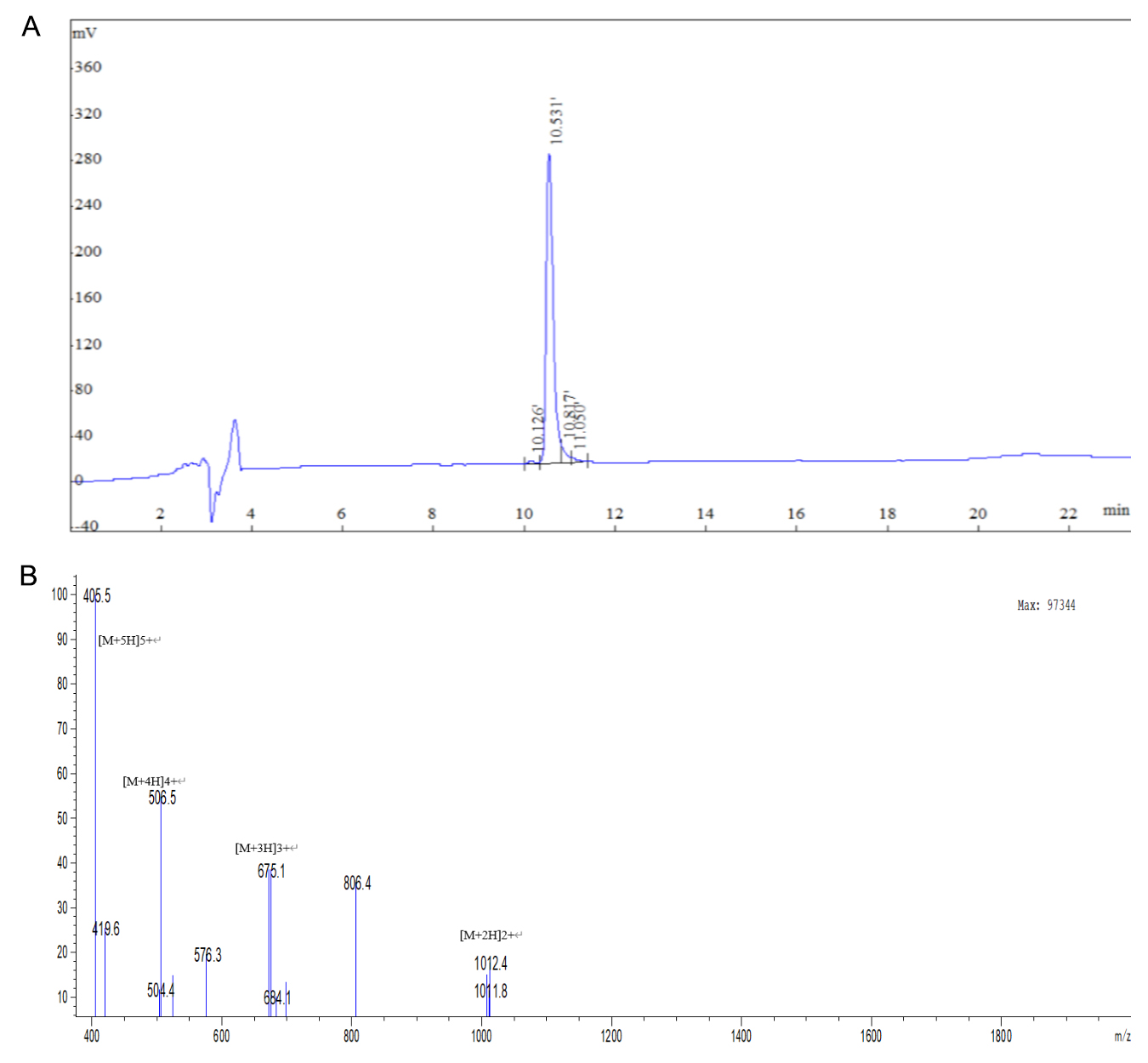

Supplement: Supplementary file 12 — Supplementary Figure S11 [file 41420_2022_1198_MOESM12_ESM.jpg]

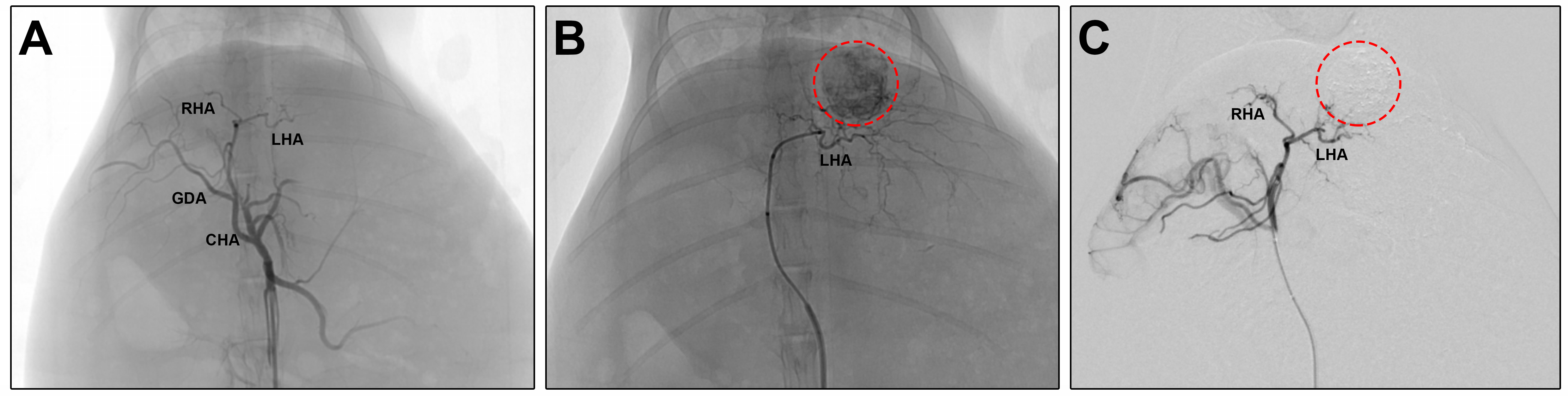

Supplement: Supplementary file 13 — Supplementary Figure S12 [file 41420_2022_1198_MOESM13_ESM.jpg]
